# Supplementary material for: Perspectives and experiences of the first geriatricians trained in Canada
Source: PLoS One. 2023 Jul 6;18(7):e0287857. doi: 10.1371/journal.pone.0287857 (PMC10325037; doi:10.1371/journal.pone.0287857)
Supplement: S1 File — (PDF) [file pone.0287857.s001.pdf]

Supporting information (Appendix)

Interview guide

An interview guide was followed for all sessions. The guide outlined the roles of the investigators and participants. The interviewer introduced the study before facilitating the discussion with the following questions:

1. How did you find yourself in geriatrics?
   a. Sub question: Why did you want to be a geriatrician?
2. What was the field of geriatrics like when you first started as a geriatrician?
   a. How does it compare to the field today?
   b. Are there aspects of the role or the system that were better or worse back then?
3. How would you compare the geriatric training you received to the training provided to geriatricians these days?
   a. What, if any, would you say are the things geriatricians are missing in their training these days?
4. What would you say is the role of a geriatrician?
   a. Do you think you have been doing this job?
   b. Is this the job that you signed up for?
   c. What would you say is the added value of a geriatrician compared to an internist when caring for older patients?
5. How do you think the field of geriatrics will change over the next 10 years?
   a. What are the biggest challenges facing the field of geriatrics over the next 10 years?
   b. What are the biggest opportunities for growth in the field of geriatrics over the next 10 years?
6. What advice would you give to a geriatrician in training?
