# Supplementary material for: Perspectives and experiences of the first geriatricians trained in Canada
Source: PLoS One. 2023 Jul 6;18(7):e0287857. doi: 10.1371/journal.pone.0287857 (PMC10325037; doi:10.1371/journal.pone.0287857)
Supplement: Attachment — Submitted filename: FirstGeri_Response to reviewer comments_29May2023.pdf [file pone.0287857.s002.pdf]

Response to reviewer comments

We thank the reviewers for their helpful comments. Our responses are below.

Comments	Author's responses
**Editor's comments**	
1. Please ensure that your manuscript meets PLOS ONE's style requirements, including those for file naming.	The manuscript has been formatted to meet PLOS ONE's requirements. We could not find instructions for file naming – please let us know if the file names are incorrect and we will correct them.
2. Please provide additional details regarding participant consent. In the ethics statement in the Methods and online submission information, please ensure that you have specified what type you obtained (for instance, written or verbal, and if verbal, how it was documented and witnessed). If your study included minors, state whether you obtained consent from parents or guardians. If the need for consent was waived by the ethics committee, please include this information.	Our study included practicing physicians; no minors were included. We have added detail about the consent in Methods section.<br><br>"Informed consent was obtained **in writing** from each participant and interviews were conducted by phone or video conference." – page 4
3. We note that the grant information you provided in the 'Funding Information' and 'Financial Disclosure' sections do not match.<br><br>When you resubmit, please ensure that you provide the correct grant numbers for the awards you received for your study in the 'Funding Information' section.	We have corrected the funding information in both locations to:<br>"The Julius and Marie Molinaro Elder Care Education Fund"
4. In your Data Availability statement, you have not specified where the minimal data set underlying the results described in your manuscript can be found. PLOS defines a study's minimal data set as the underlying data used to reach the conclusions drawn in the manuscript and any additional data required to replicate the reported study findings in their entirety. All PLOS journals	We have carefully considered the data sharing requirement. The main issue is that the participants (Canada's first geriatricians) is a very small group. Their accounts and experiences are relatively unique, making the transcripts readily identifiable by certain audiences, even with deidentification. Some of the accounts are personal, so we have not received consent for release of the transcripts.

require that the minimal data set be made fully available. For more information about our data policy, please see http://journals.plos.org/plosone/s/data-availability.<br><br>"Upon re-submitting your revised manuscript, please upload your study's minimal underlying data set as either Supporting Information files or to a stable, public repository and include the relevant URLs, DOIs, or accession numbers within your revised cover letter. For a list of acceptable repositories, please see http://journals.plos.org/plosone/s/data-availability#loc-recommended-repositories. Any potentially identifying patient information must be fully anonymized.<br><br>Important: If there are ethical or legal restrictions to sharing your data publicly, please explain these restrictions in detail. Please see our guidelines for more information on what we consider unacceptable restrictions to publicly sharing data: http://journals.plos.org/plosone/s/data-availability#loc-unacceptable-data-access-restrictions. Note that it is not acceptable for the authors to be the sole named individuals responsible for ensuring data access.<br><br>We will update your Data Availability statement to reflect the information you provide in your cover letter.	However, we would be able to provide data to support the manuscript based on reasonable requests. For data requests, authors could contact the Research Ethics Board at Unity Health Toronto:<br><br>Sharon Freitag<br>Senior Director, Research Ethics<br>Unity Health Toronto<br>Phone: 416.864.6060 ext. 42385<br>Email: sharon.freitag@unityhealth.to
5. Please review your reference list to ensure that it is complete and correct. If you have cited papers that have been retracted, please include the rationale for doing so in the manuscript text, or remove these references and replace them with relevant current references. Any changes to the reference list should be mentioned in the rebuttal letter that accompanies your revised manuscript. If you need to cite a retracted article, indicate the	The reference list is correct and none of the articles have been retracted to our knowledge.

article's retracted status in the References list and also include a citation and full reference for the retraction notice.	
**Reviewer's comments**	
Reviewer #1: This manuscript- despite being an easy read -addresses a very important perspective to young physicians considering a geriatric specialty. It also may help physicians thinking about establishing a geriatric specialty in their countries (Geriatrics is not yet present in many countries).	Thank you.
Authors stated that data are not fully disclosed and are not being shared publicly. Is it possible to share after removing any personal details?	We have considered this. Since the pool of original geriatricians is small, their accounts and encounters are relatively unique. Even if we removed personal identifiers, their experiences alone may allow them to be identified. We have therefore decided not to release the original transcripts, some of which contain very personal information. See data sharing response above.
The manuscript is well-written. Apart from a typo in table 1 section about advice for trainees- distance them themselves, there are no other remarks.	Thank you. We corrected the error. The line from table 1 now reads:<br><br>"I would encourage [trainees] to not distance themselves too far from acute care, because that really keeps you up on your internal medicine." – Participant 8
Reviewing appendix 1: the questionnaire included a question about what the participants found something missing from the training of current geriatric trainees, this was not mentioned in the manuscript and could provide meaningful insight to anyone involved in the establishment of training programs for geriatricians.	Added line in results:<br><br>"Participants also noted gaps in the training programs included limited community practice exposure and administrative management skills." – page 11<br><br>Also added 2 quotes in Table 1.<br>"Gaps in current training programs" under "the training process"<br><br>"I think the biggest gap is how to set up a practice. It depends where you are going to practice, but if you are going to community, it

	takes a lot to carve out the resources that you need… And I'm not sure how well we provide trainees with the [knowledge] to do that." – Participant 3<br><br>"There are those of us who come to medicine with a different background. I wonder if [we are] losing out on applicants who've had challenges in their life, and I'm not just talking financial [challenges], but academic or otherwise who actually would make amazing physicians, but they don't get the opportunity because their CV just doesn't match that template… That's sometimes how I feel when I look at the applicants: I see very accomplished individuals… but I'm not sure how much diversity we are attracting." – Participant 6
Reviewer #2: This a very interesting study aiming to examine the perspectives and experiences of the first cohorts of geriatricians in Canada. Through structured interviews experienced geriatricians told their impressions of the specialty. The main topics discussed were the role of advocacy for older people and geriatrics as the less taken road.	
In the introduction, you could complement it by adding evidence on the subject from Canada or elsewhere. Is there any information on perspectives from geriatricians? Show there is a gap of knowledge, and why this would be relevant to know.	We have added the following line to introduction:<br><br>"Despite this, the perspectives of geriatrician pioneers have rarely been reported in a qualitative study." – page 3<br><br>We could not find any similar studies interviewing geriatricians directly on their career and perspectives.
In the discussion, it might be worth it to compare the reasons to choose geriatrics mentioned by the respondents with younger geriatricians. Also is there any evidence that geriatricians can decrease ageism?	We could not find data to support a geriatrician's role in reducing ageism and we agree this is an important future research direction. We also agree with the point about seeking perspectives from younger geriatricians.

	We have added the following to the manuscript:<br><br>"However, studies investigating the role of geriatricians in reducing ageism are lacking." Page 15<br><br>Under limitations, we have added:<br>"We also did not include younger geriatricians to determine if their experiences are different, a topic which could be addressed in future studies." – Page 16
